# Alantolactone, a natural sesquiterpene lactone, has potent antitumor activity against glioblastoma by targeting IKKβ kinase activity and interrupting NF-κB/COX-2-mediated signaling cascades

**DOI:** 10.1186/s13046-017-0563-8

**Published:** 2017-07-12

**Authors:** Xun Wang, Zhenlong Yu, Chao Wang, Wei Cheng, Xiangge Tian, Xiaokui Huo, Yan Wang, Chengpeng Sun, Lei Feng, Jinshan Xing, Yulong Lan, Dongdong Sun, Qingjuan Hou, Baojing Zhang, Xiaochi Ma, Bo Zhang

**Affiliations:** 1 Department of Neurosurgery of the Second Affiliated Hospital, College of Pharmacy, Institute of Cancer Stem Cell, Dalian Medical University, Dalian, China; 2 Department of Neurosurgery, the Third People’s Hospital of Dalian, Non-directly Affiliated Hospital of Dalian Medical University, Dalian, China

**Keywords:** Alantolactone, Glioblastoma multiforme, Blood-brain barrier, Cox-2, IKKβ/NF-κB

## Abstract

**Background:**

Glioblastoma multiforme (GBM) is one of the most refractory and palindromic central nervous system (CNS) neoplasms, and current treatments have poor effects in GBM patients. Hence, the identification of novel therapeutic targets and the development of effective treatment strategies are essential. Alantolactone (ATL) has a wide range of pharmacological activities, and its anti-tumor effect is receiving increasing attention. However, the molecular mechanism underlying the anti-GBM activity of ATL remains poorly understood.

**Methods:**

The biological functions of ATL in GBM cells were investigated using migration/invasion, colony formation and cell cycle/apoptosis assays. The localization of nuclear factor kappa B (NF-κB) p50/p65 and its binding to the cyclooxygenase 2 (COX-2) promoter were determined using confocal immunofluorescence, a streptavidin-agarose pulldown assay and a chromatin immunoprecipitation (ChIP) assay. IKKβ kinase activity was determined using a cell IKKβ kinase activity spectrophotometry quantitative detection kit and a molecular docking study. LC-MS/MS analysis was performed to determine the ability of ATL to traverse the blood-brain barrier (BBB). The in vivo anti-tumor efficacy of ATL was also analyzed in xenografted nude mice. Western blot analysis was performed to detect the protein expression levels.

**Results:**

ATL significantly suppressed the growth of GBM in vivo and in vitro. ATL significantly reduced the expression of COX-2 by inhibiting the kinase activity of IKKβ by targeting the ATP-binding site and then attenuating the binding of NF-κB to the COX-2 promoter region. Furthermore, ATL induced apoptosis by activating the cytochrome c (cyt c)/caspase cascade signaling pathway. Moreover, ATL could penetrate the BBB.

**Conclusions:**

ATL exerts its anti-tumor effects in human GBM cells at least in part via NF-κB/COX-2-mediated signaling cascades by inhibiting IKKβ kinase activity. ATL, which is a natural small molecule inhibitor, is a promising candidate for clinical applications in the treatment of CNS tumors.

## Background

Gliomas are the most common primary tumors in the central nervous system (CNS), accounting for approximately 60 to 70% of primary brain tumors [1]. Glioblastoma multiforme (GBM) represents the highest grade of malignancy. The current treatment for GBM relies on surgical resection followed by radiotherapy combined with chemotherapy. Unfortunately, these treatments have poor effects in GBM patients, and the median survival is only 12 months [2]. Therefore, new therapeutic strategies are urgently needed. Molecular targeted therapy has become a hot topic in the study of the molecular mechanisms underlying GBM resistance [3, 4]. However, the blood-brain barrier (BBB) restricts the application of many drugs. Thus, the identification of a drug that could act on new therapeutic targets and, more importantly, penetrate the BBB could benefit many GBM patients.

Recently, increasing attention has been paid to chronic inflammation, such meningitis-associated malignant brain tumor, which has been shown to increase the risk of cancer [5–8]. The inflammatory microenvironment, which is a basic characteristic of malignant tumors, is involved in the modulation of the tumor viability, migration, invasion, and even inflammation [9–11]. Therefore, strategies focused on inhibiting the inflammatory reaction using specific small molecule inhibitors could offer significant therapeutic value in the treatment of malignant tumors.

Cyclooxygenase 2 (COX-2) is associated with inflammatory diseases, carcinogenesis [9, 12] and resistance to apoptosis [13, 14], suggesting that COX-2 may play important roles by mediating the protective effects of inflammation. In addition, COX-2 is a target of non-steroidal anti-inflammatory drugs (NSAIDs), and its selective inhibitors could effectively prevent inflammation, proliferation and angiogenesis and induce apoptosis in human cancer cells. The overexpression of COX-2 has been found to be important in the development of several human tumor types, including gliomas [15, 16], and has been associated with a high tumor aggressiveness and a poor prognosis in patients [17, 18]. In intracranial tumors, the enhanced expression of COX-2 is correlated with the histopathological grade of the gliomas [19]. Therefore, the inhibition of the expression of COX-2 might be an effective alternative approach for suppressing glioblastoma growth.

The expression of COX-2 is strictly and specifically controlled by the binding of many trans-factors, such as activator protein-2 (AP-2) and nuclear factor kappa B (NF-κB) [20, 21], and certain transcriptional coactivators, such as p300 [22, 23], to corresponding sites on its promoters. The activation of the NF-κB signaling pathway was involved in the overexpression of COX-2 [24, 25]. Canonical NF-κB activation depends on the degradation of IκB, which is rapidly phosphorylated by an active IκB kinase (IKK) complex. This complex is composed of IKKα and IKKβ catalytic subunits and a regulatory subunit, i.e., IKKγ/NEMO (NF-κB essential modulator) [26]. However, IKKβ is the major subunit that is responsible for the phosphorylation of the IκB proteins. The phosphorylated IκB subsequently undergoes proteasome-mediated degradation, thereby liberating free NF-κB dimers and allowing these dimers to translocate to the nucleus and promote gene transcription [27]. Thus, the identification of small molecule inhibitors that selectively target IKKβ and an understanding of the mechanisms regulating the activation of NF-κB are essential.

Alantolactone (ATL), which is a natural sesquiterpene lactone, is largely distributed in the medical herb *Inula helenium* and possesses a wide range of biological activities, such as antibacterial, antifungal, anti-inflammatory and hepatoprotective activities [28], as detailed in the records of the China Pharmacopoeia and European Pharmacopoeia. ATL has a rapid onset and does not cause significant damage to normal animal tissues and organs [29, 30]. The antitumor properties of ATL have been demonstrated in peripheral tumors, including lung cancer, liver cancer, colon cancer, and leukemia [31–35]. However, to date, the detailed anti-cancer and anti-inflammatory mechanisms by which ATL exerts its effects have not been characterized. Furthermore, ATL, which is a small molecule of volatile oil compounds, is consistent with the traditional Chinese Medicine theory of “upward into the brain” and has a great potential to permeate the BBB.

In this study, we investigated whether ATL inhibits glioblastoma growth by suppressing the expression of COX-2 both in vitro and *vivo*. In addition, the molecular effects of ALT on glioblastomas was investigated by assessing the changes in the NF-κB signaling pathway. Furthermore, we also assessed ATL levels in the cerebrospinal fluid using a rat model, which confirmed that ATL was able to cross the BBB. Therefore, ATL has potential applications in the treatment of CNS tumors.

## Methods

### Transwell invasion assay

Cell invasion was analyzed using a Transwell assay [36]. U87 and U251 cells were plated in 24-well Transwell plates. The upper surface of the polycarbonate filters was coated with Matrigel and incubated for 1 h at 37 °C for gelling. The cells (5 × 10 [4]) were seeded into the upper chambers in FBS-free DMEM, and the bottom chambers were filled with 600 μL of DMEM with 10% FBS. Both the top and bottom chambers contained the same concentrations of ATL. After 24 h of incubation, the non-invasive cells on the upper membrane surfaces were removed by wiping with cotton swabs. The invading cells were fixed with methanol and stained with a 0.1% Crystal Violet staining solution. Images were taken under a Leica DM 14000B microscope. Cell invasion was counted in five independent areas per membrane. The results are represented as the means calculated from five replicates of each experiment.

### Flow cytometry analysis

To determine the distribution of the cells in the cell cycle and the proportion of apoptotic cells, we performed flow cytometry analysis using a flow cytometer (BD FACS Accuri C6, CA, USA). After a 24 h treatment with ATL (0, 10 and 20 μM), the cells were collected, washed with PBS and fixed with ice-cold 70% ethanol at 4 °C for 4 h. The cells were stained with propidium iodide (PI) staining buffer (0.2% Triton X-100, 100 μg/mL DNase-free RNase A, and 50 μg/mL PI in PBS) in the dark for 30 min. For the apoptosis examination, the cells were washed with PBS, collected, and stained using the Annexin V-FITC Apoptosis Detection Kit in the dark at room temperature for 15 min. The cell cycle distribution and the fraction of apoptotic cells were determined using a FACS analysis system. Each experimentwas performed in triplicate.

### Western blot analysis

The cell lysate proteins were separated by electrophoresis on a 7.5-12% SDS-PAGE and probed with specific antibodies. The protein bands were detected by enhanced chemiluminescence. The protein concentrations were determined using a BCA protein assay kit (Beyotime Biotechnology, China). Similar experiments were performed at least three times.

### Reverse-transcriptase polymerase chain reaction (RT-PCR)

Total RNA was extracted from ATL-treated U87 and U251 cells using the TRIzol reagent, according to the kit protocol (TaKaRa Bio, Dalian, China). The cDNA was reverse-transcribed using the PrimeScript RT Reagent Kit (TaKaRa Bio, Dalian, China), according to the manufacturer’s instructions. The primer pairs were as follows: COX-2, Forward: 5′-TCACAGGCTTCCATTGACCAG-3 and Reverse: 5′-CCGAGGCTTTTCTA CCAGA-3′; β-actin, Forward: 5′-GGCACCCAGCACAATGAA-3′ and Reverse: 5′-TAGAAGCATTTGCGGTGG −3′. The amplification products were analyzed using a 1.5% agarose gel electrophoresis, stained with ethidium bromide, and photographed under ultraviolet light.

### Confocal immunofluorescence

Briefly [37], ATL-treated U87 cells were grown on chamber slides, fixed with 4% paraformaldehyde and permeabilized with 0.2% TritonX-100. The samples were probed with specific antibodies against Cytochrome c (cyt c), p300, p50 or p65 (Santa Cruz) and fluorescein isothiocyanate- and rhodamine-conjugated secondary antibodies Subsequently, the stained samples were mounted with 4′, 6-diamidino-2-phenylindole (DAPI) to counterstain the cell nuclei. After five additional 5-min washes, the samples were examined under a Leica DM 14000B confocal microscope.

### Streptavidin-agarose pulldown assay to detect DNA protein binding

The binding assay was performed by mixing 400 μg of the nuclear extract proteins, 4 μg of the biotinylated DNA probe and 40 μl of 4% streptavidin-conjugated agarose beads at RT for 5 h in a rotating shaker. The beads were centrifuged, resuspended with the SDS-PAGE loading buffer and boiled at 95 °C. The supernatant was analyzed by Western blotting.

### Chromatin immunoprecipitation (ChIP)

The ChIP assay was performed as previously described [37]. The specific COX-2 promoter primers were as follows: forward primer: ACGTGACTTCCTCGACCCTC, and reverse primer: AAGACTGAAAA CCAAGCCCA). The resulting 478 bp product of COX-2 was separated by 1.5% agarose gel electrophoresis.

### IKKβ kinase activity assay in vitro

ATL-mediated inhibition of IKKβ kinase activity was assessed in vitro using a cell IKKβ kinase activity spectrophotometry quantitative detection kit. Briefly, ALT-treated U87 cells were harvested and lysed with the lysate in the kit. After the protein was quantified, 10 μl of the sample solution (containing 50 μg of protein) was mixed with the reaction solution in the kit. The total activity and nonspecific activity were measured using a microplate reader. The data were evaluated according to the formula in the manual, and the specific activity value was calculated (specific activity = total activity - nonspecific activity).

### Molecular modeling

Docking studies were performed to explore the potential binding mode between ATL and the IKKβ protein complex. ATL was optimized using the semi-empirical PM3 method with the Polak-Ribie’re conjugate gradient algorithm and an RMS gradient of 0.01 kcal mol − 1 Å − 1 as the convergence criterion. The optimized structure of ATL was docked to the active site of IKKβ with ligand K-252A (PDB Code: 4KIK). The crystallographic ligand was extracted from the active site, and the residues within a 6.5 A° radius around the IKKβ molecule were defined as the active pocket. The SurflexDock program was used for the docking calculations with the default parameters. MOLCAD surfaces were generated to visualize the binding mode of the docked protein–ligand complexes.

### Animal studies

Male nude mice (BALB/c nu/nu, 4 weeks old, 18–19 g) were purchased from the SPF Laboratory Animal Center of Dalian Medical University (Dalian, China). Briefly, 1 × 10^7^ U87 cells were injected subcutaneously near the axillary fossa of the nude mice. The tumor cell–inoculated mice were randomly divided into the following three treatment groups with five mice in each group: group A was treated with propylene glycol; group B was treated with 10 mg/kg ATL; and group C was treated with 20 mg/kg ATL; all treatments were delivered by daily intraperitoneal injections. The tumors were measured using a caliper every 2 days, and the tumor volume was calculated according to the formula V = 1/2 (width [2]× length). The body weights were also recorded. After treatment with ATL for 15 days, all experimental mice were terminated with ether anesthesia, and the total weight of the tumors in each mouse was measured. To determine the expression of COX-2 and p65 NF-κB, the tumor tissues were fixed with 10% neutral formalin and embedded in paraffin. The sections (4 μm) were stained with the COX-2 antibody (1:50) and the p-p65 NF-κB (1:50) antibody and examined under a light microscope. The images were examined under a Leica DM 4000B microscope equipped with a digital camera.

All animals were given free access to sterilized food and water and were habituated for 7 days before the experiments. All procedures were in accordance with the National Institutes of Health Guide for the Care and Use of Laboratory Animals (National Institutes of Health, Bethesda, MD, USA). The protocol was approved by the Animal Care and Ethics Committee of Dalian Medical University.

### Detection of ATL through the BBB

Six male adult SD rats (200-220 g) were intraperitoneally injected with ATL; after 1 h, the rats were anesthetized with 4% chloral hydrate. Cerebrospinal fluid (50-100 μl) was collected from the cerebellomedullary cistern by puncturing the foramen magnum. Then, the cerebrospinal fluid was extracted twice using an equal volume of acetonitrile. The supernatant was dried in a nitrogen blowing instrument and reconstituted in 50 μl mobile phase (acetonitrile: pure water = 45:55). Finally, the reconstituted sample and ATL standard solution were analyzed by LC-MS/MS.

### Statistical analysis

The data are represented as the mean ± SD of at least three independent experiments. An analysis of variance and Student’s t-test were used to compare the values of the test and control samples in vitro and in vivo. *P* < 0.05 was considered statistically significant. SPSS 18.0 software was used for all statistical analyses.

## Results

### ATL alters cell morphology and inhibits cell viability in human glioblastoma cells

**Fig. 1:**
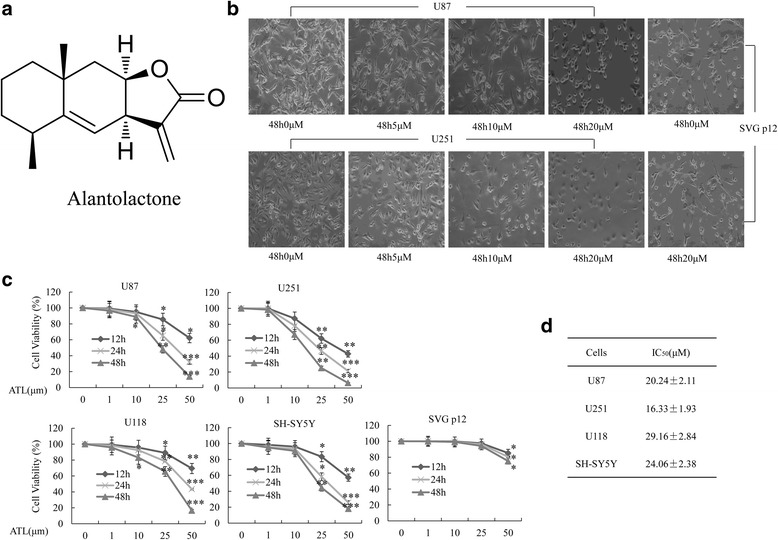
ATL changes morphology and inhibits cell viability. **a**: The chemical structure of ATL. **b**: Changes in the cell morphology and proliferation of U87, U251 and SVG p12 cells were observed after treatment with ATL for 48 h (original magnification, 200×). **c**: U87, U251, U118, SHSY-5Y and SVG p12 cells were cultured with the indicated concentrations of ATL for the indicated hours; then, MTT assays were performed. **d**: At 48 h after treatment, cell viability was determined using MTT assays in the indicated cell lines, and the IC_50_ value was calculated. The data are shown as the mean ± SD. *P < 0.05, **P < 0.01, vs. the DMSO-treated group

To determine whether ATL could inhibit glioblastoma cell proliferation, we first assessed the changes in the cell morphology and proliferation of U87 and U251 cells. As shown in Fig. 1b, shrunken cells and plasma membrane blebs were observed after treatment with ATL. Then, we quantitatively analyzed the effect of ATL on cell proliferation in U87, U251, U118 and SH-SY5Y cells using an MTT assay. As shown in Fig. 1c, the survival rate of the four cell lines was reduced in a dose- and time-dependent manner after treatment with increasing concentrations of ATL (0, 1, 10, 25 and 50 μM) for 12, 24 and 48 h. The IC_50_ value of ATL was 20.24 ± 2.11 μM (U87), 16.33 ± 1.93 μM (U251), 29.16 ± 2.84 μM (U118), and 24.06 ± 2.38 μM (SH-SY5Y) after 48 h of treatment (Fig. 1d). Interestingly, ATL showed no obvious cytotoxicity at low concentrations in the normal human glial cell line (SVG p12, Fig. 1b and c).

### ATL inhibits colony formation and cell cycle arrest

**Fig. 2:**
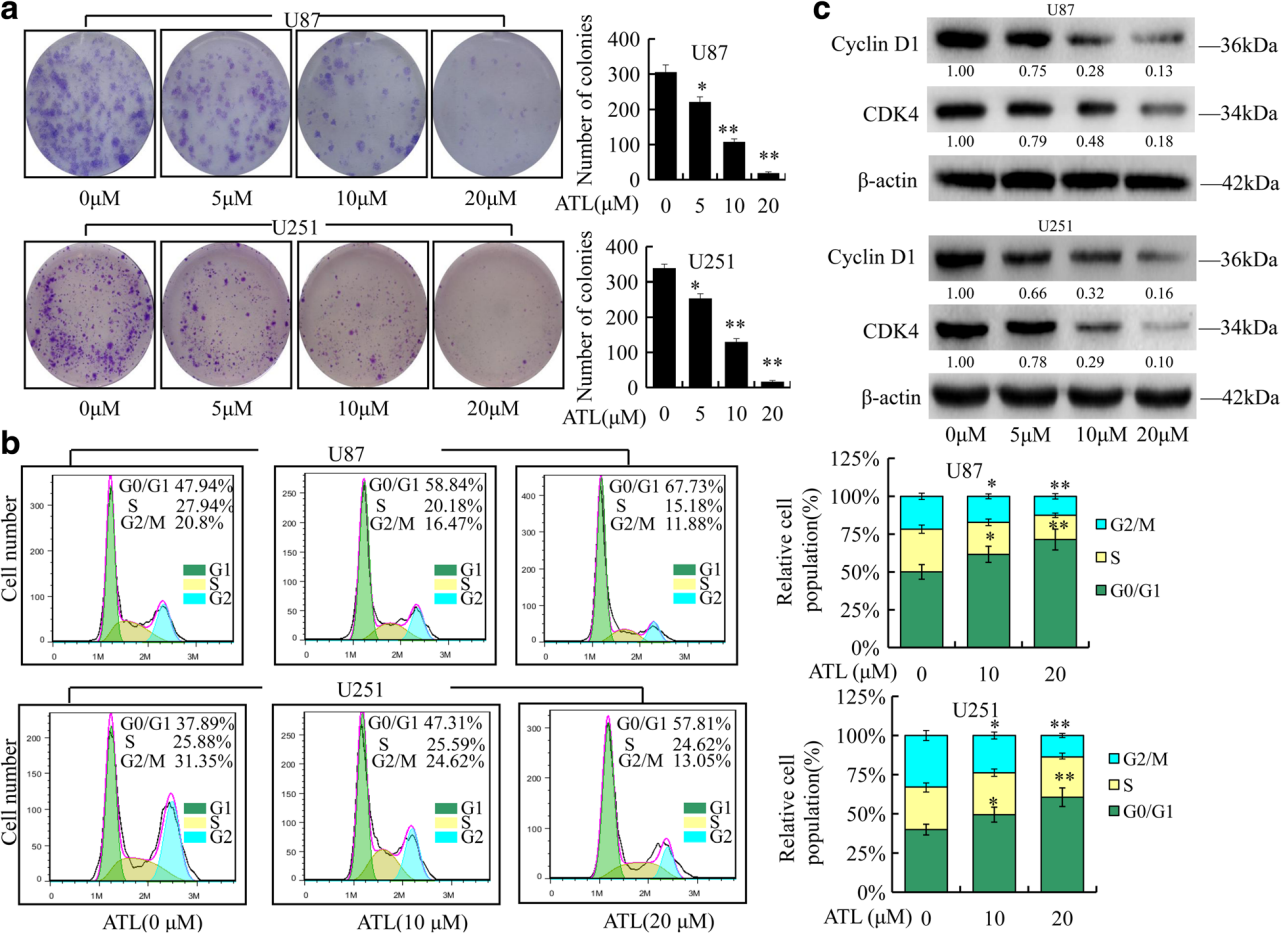
ATL inhibits cell colony formation and blocks the cell cycle. **a**: U87 and U251 cells were treated with ATL at the indicated doses for the appropriate time; then, the induced colony formation was analyzed, and the colony formation numbers were calculated. **b**: U87 and U251 cell cycle analysis was performed using a C6 flow cytometer after 48 h of ATL treatment. **c**: Cyclin D1 and CDK4, which are cell cycle-related proteins in the G1 phase, were evaluated by Western blotting in the two cell lines. The data are presented as the mean ± SD of three separate experiments (*P < 0.05, **P < 0.01, significant differences between the ATL treatment groups and DMSO vehicle control groups)

A clonogenic cell survival assay was performed to evaluate the influence of ATL on the clonogenic capacity of U87 and U251 cells. As shown in Fig. 2a, treatment with ATL markedly inhibited colony formation and resulted in a significant decrease in the colony formation ratio. Cell proliferation inhibition often accompanies changes in cell cycle progression [38]. We then evaluated the impact of ATL on cell cycle arrest. Compared with the control group, the percentages of cells in the G0/G1 phase increased from 47.94 to 67.73% and 37.89 to 57.81% after ATL in U87 and U251 cells, respectively, whereas the percentages of cells in the G2/M phase decreased from 20.8 to 11.88% and 31.35 to 13.05%, respectively (Fig. 2b). Thus, ATL inhibits the proliferation of U87 and U251 cells by inducing cell-cycle arrest at the G0/G1 phase.

Furthermore, to ascertain the detailed mechanisms underlying ATL-induced cell cycle arrest, several cell cycle-related proteins, including cyclin D1 and CDK4, were evaluated by Western blotting (Fig. 2c). The loss of these proteins’ expression might be attributed to the cell cycle arrest.

### ATL inhibits cell migration and inhibition

**Fig. 3:**
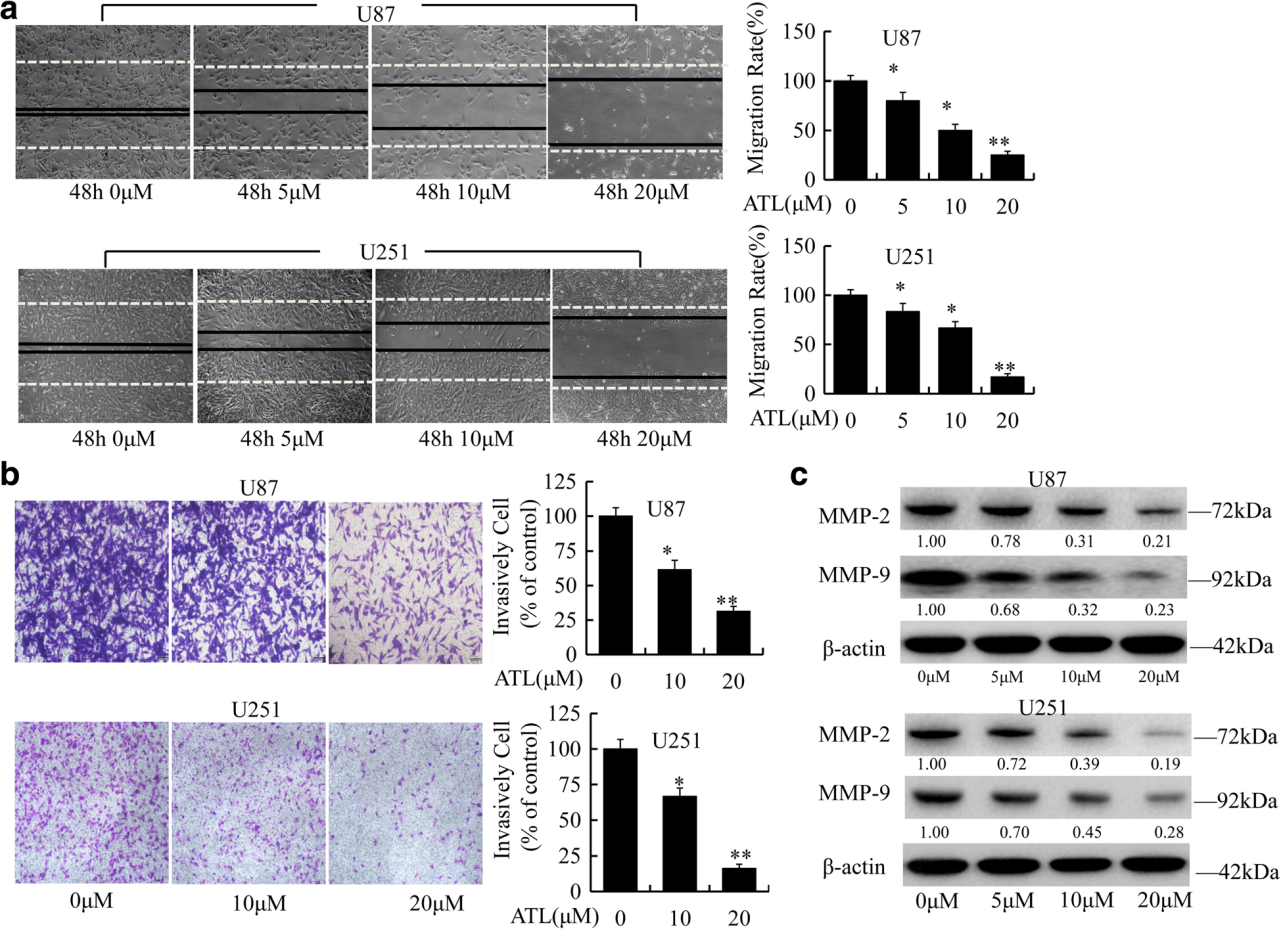
ATL inhibits cell migration and invasion. **a**: Cell migration was analyzed using a wound-healing assay in U87 and U251 cells as described in the “Materials and Methods” section (original magnification, 100×); then, the migration rate was calculated. **b**: ATL suppresses cell invasion in the Transwell assay. The two cell types were plated in Matrigel pre-coated Transwell chamber. Cells that have passed through to the bottom of the membrane were counted (original magnification, 100×). **c**: MMP-2 and MMP-9, which are invasive marker proteins, were detected by Western blotting in the two cell lines. Three independent experiments were performed. *P < 0.05, **P < 0.01, vs. the DMSO-treated group

We performed wound healing assays and Transwell assays to determine the effects of ATL on cell migration and invasion in glioblastoma cells. As shown in Fig. 3a and b, ATL significantly inhibited the migration and invasion of both U87 and U251 cells in a dose-dependent manner. We also examined the expression level of several cell migration- and invasion-related proteins, including the matrix metalloproteinases (MMPs), and found that ATL could also obviously inhibit the expression of MMP-2 and MMP-9 (Fig. 3c). Thus, ATL possesses the properties required for suppressing the cell migration and invasion of glioblastoma cells.

### ATL induces apoptosis by modulating cyt c and caspase signaling

**Fig. 4:**
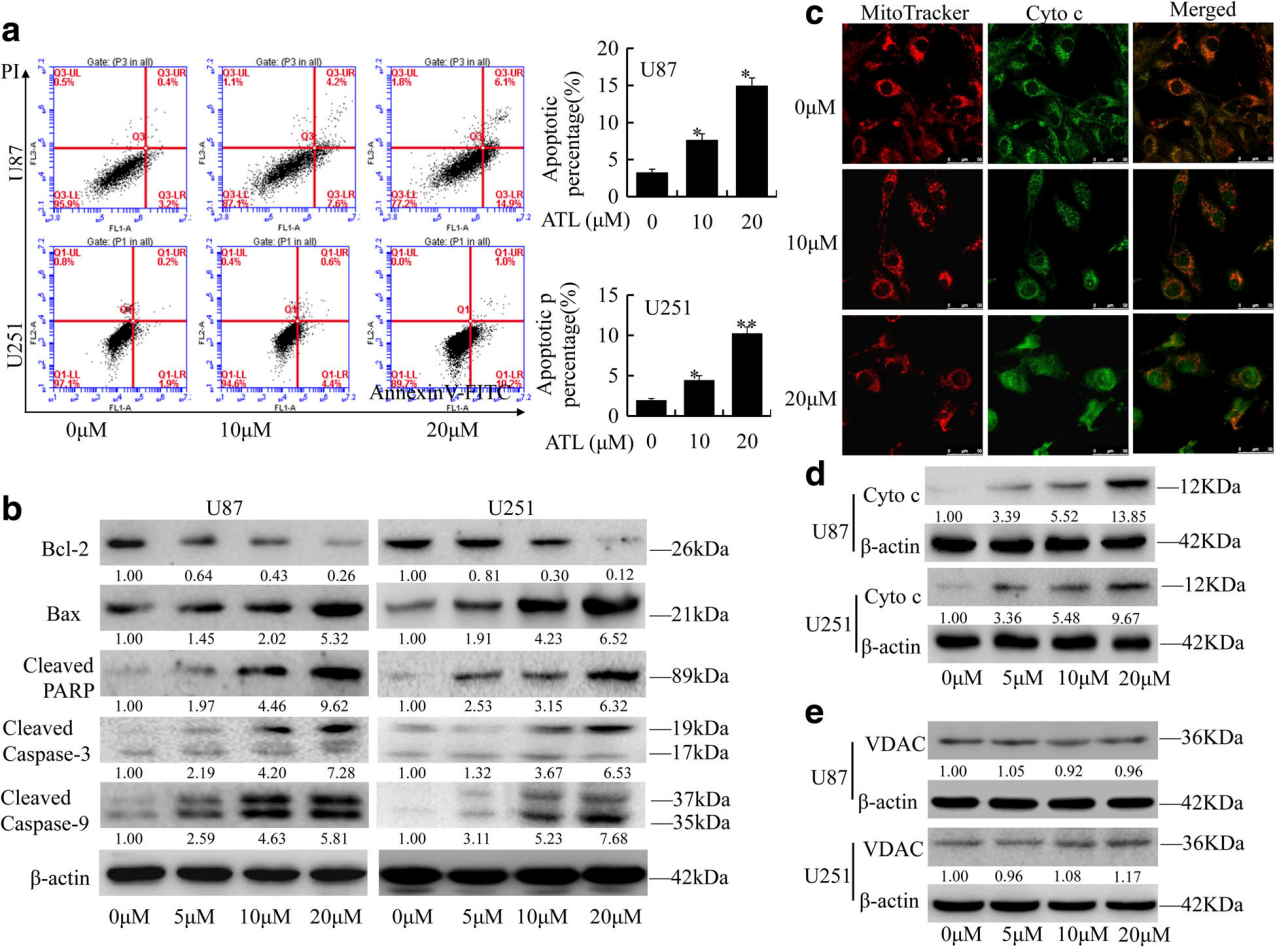
ATL induces apoptosis by modulating cyt c/caspase signaling. **a**: Apoptosis was analyzed using FACS. The results are represented as the mean ± SD of three experiments. *P < 0.05, **P < 0.01 vs. the DMSO-treated group. **b**: The indicated pro-apoptotic and anti-apoptotic proteins were detected by a Western blot analysis. **c**: The release of cyt c from the mitochondria to the cytoplasm was observed via am IFI analysis in U87 cells. **d**: The protein expression of cyt c in the cytoplasm of these two cell types was detected by a Western blotting assay. **e**: The protein levels of the VDAC were detected by Western blotting in U87 and U251 cells. The results are represented as the mean ± SD of three experiments. *P < 0.05, **P < 0.01 vs. the DMSO-treated group. The experiment was repeated three independent times

We then examined whether ATL-induced cell growth inhibition was associated with an increase in apoptosis in glioblastoma cells. ATL treatment resulted in a significant dose-dependent induction of apoptosis in both U87 and U251 cells (Fig. 4a). To ascertain the detailed mechanisms underlying ATL-induced cell apoptosis, several pro-apoptotic and anti-apoptotic proteins were also detected by Western blot analysis, including caspase-3/9, PARP, BAX and Bcl-2, in the two cell lines. As shown in Fig. 4b, compared with the control group, ATL could markedly increase the expression levels of cleaved caspase-3/9, cleaved PARP and the BAX proteins but decreased the protein levels of Bcl-2. Thus, ATL may function as an important and specific mediator that facilitates the activation of multiple caspase cascades.

Moreover, cyt c is released from the mitochondria into the cytosol and could induce apoptosis. We then performed an immunofluorescence imaging (IFI) analysis to confirm the co-localization of cyt c and mitochondria to determine whether ATL treatment could induce the release of cyt c. As shown in Fig. 4c, treatment with ATL (10 μM and 20 μM) markedly triggered the release of cyt c from the inter-mitochondrial space into the cytosol. Meanwhile, cyt c was also detected in the cytoplasm, where the mitochondrial fraction was isolated (Fig. 4d). To exclude the possibility that the amount of mitochondria has changed, we also perform the WB analysis of VDAC and found that ATL treatment had no obvious changes on mitochondria (Fig. 4e). Thus, ATL could induce apoptosis in glioblastoma cells by triggering the release of cyt c and facilitating the activation of caspase in the cytosol.

### ATL inhibits COX-2 signaling in human glioblastoma cells

**Fig. 5:**
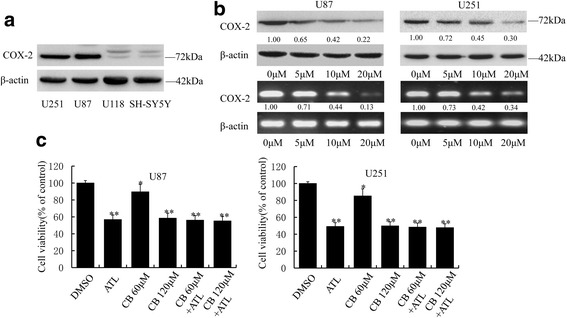
ATL inhibits the expression of COX-2. **a**: We first observed the expression of COX-2 in four nerve tumor cell lines by Western blotting assays. **b**: At 48 h after treatment, the expression levels of the COX-2 protein and mRNA were detected by Western blotting and RT-PCR, respectively, in U87 and U251 cells. **c**: The two cell types were pretreated with the COX-2-selective inhibitor celecoxib (CB, 60 μM and 120 μM)) for 8 h and then treated with ATL (20 μM). After 48 h, cell viability was observed using MTT analysis. All data are presented as the mean ± SD of three independent experiments (*P < 0.05, **P < 0.01 vs. the DMSO vehicle control groups)

High expression of COX-2 is implicated in the cell growth, migration and invasion of cancer cells [39–42]. To determine the influence of ATL on COX-2 signaling, COX-2 protein and gene levels in ATL-treated cancer cells were assessed by RT-PCR and Western blotting. We first assessed the expression of COX-2 in nerve tumor cells, including U251, U87, U118 and SHSY-5Y cells. As shown in Fig. 5a, COX-2 protein was more abundant in U251 and U87 cells than in U118 and SHSY-5Y cells, COX-2 protein was correlated with the histopathological grade of the gliomas. Treatment with ATL significantly inhibited the expression of COX-2 at both the protein and mRNA levels in U87 and U251 cells in a dose-dependent manner (Fig. 5b). Then, U251 and U87 cells were pretreated with the COX-2-selective inhibitor CB (60 μM and 120 μM) for 8 h, followed by ATL (20 μM). After a continuous 48 h incubation, cell viability was analyzed using an MTT assay. As shown in Fig. 5c, treatment with CB or ATL alone inhibited cell proliferation, whereas the combination of CB and ATL did not significantly alter the inhibition of cell viability. ATL-mediated inhibition of the proliferation of glioblastoma cells is mediated at least in part by the ability of ATL to inactivate COX-2 signaling.

### ATL inhibits the translocation of NF-κB and p300 and the binding of these proteins to the COX-2 promoter

**Fig. 6:**
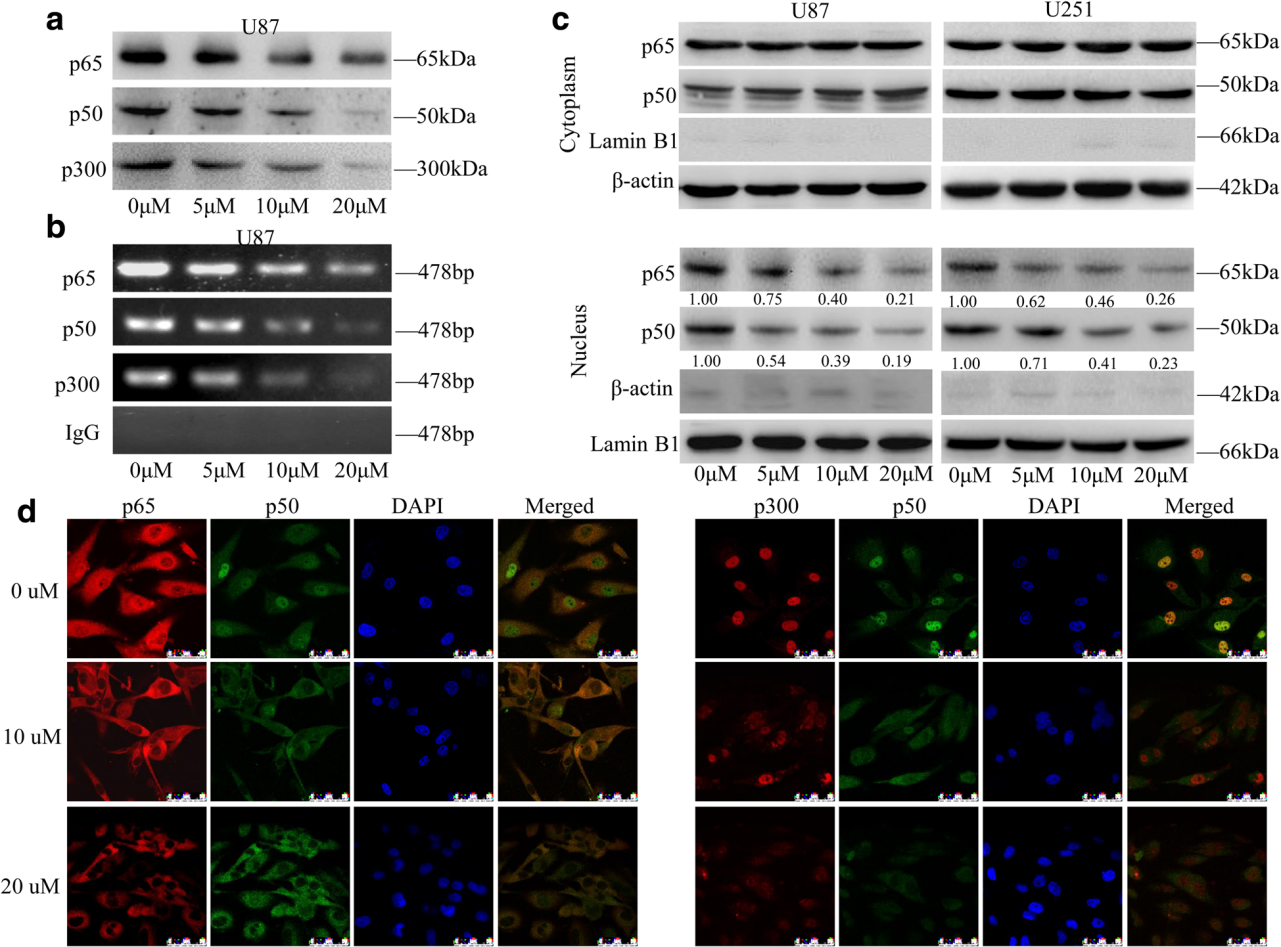
ATL suppresses the translocation of NF-κB p65/p50 and p300 and the binding of these proteins to the COX-2 promoter. **a**: After 48 h of ATL treatment, we assessed the binding of p300, p65 and p50 to the COX-2 promoter probe via streptavidin-agarose pulldown assays in U87 cells. **b**: Correspondingly, chromatin was immunoprecipitated with antibodies against p65, p50 and p300 after treatment with ATL for 48 h; then, we determined the levels of the COX-2 promoter region in the precipitated chromatin by RT-PCR. **c**: We first separated the cytoplasmic and nuclear proteins and then assessed the expression of p65 and p50 by Western blot analysis with β-actin and Lamin B1 as controls for the sample loading. **d**: At 48 h after treatment, we also observed the subcellular localization of p65, p50, and p300 and the colocalization of p50 with p65 or p300 by a confocal microscopy analysis

ATL effectively suppressed the gene expression of COX-2; however, the gene expression of COX-2 was regulated by several transcription factors, including NF-κB and p300, and a transcriptional coactivator in its promoter region [43]. Then, we performed a streptavidin-agarose pulldown assay to evaluate the effect of ATL on the binding activities of NF-κB and p300 in the COX-2 promoter using a 478-bp biotin-labeled double-stranded oligonucleotide probe corresponding to the 5′-flanking sequence of the COX-2 gene from −30 to −508 [44]. Compared with the control treatment, ATL markedly inhibited the binding of the NF-κB p50 and p65 subunits to the COX-2 promoter DNA probe (Fig. 6a) in a dose-dependent manner. To further confirm this binding inhibition, we performed a ChIP assay using specific antibodies. As shown in Fig. 6b, compared with the control treatment, ATL also markedly increased the inhibition of the binding of NF-κB p50 and p65 to the chromatin COX-2 promoter. Moreover, ATL treatment dose-dependently inhibited the binding of the co-activator p300 to the COX-2 promoter (Fig. 6a and b). We also detected the protein levels of the NF-κB p50/p65 subunits in whole-cell lysates and nuclear lysates. As shown in Fig. 6c, the expression of p65/p50 in the nucleus significantly decreased after ATL treatment. To exclude the possibility of the contaminations between cytoplasmic and nuclear fractions, we also check the expression of lamin B1 in the cytoplasmic fraction and beta-actin in the nuclear fraction. No obvious contaminations were found (Fig. 6c). Based on these results, we hypothesized that treatment with ATL markedly inhibited the translocation of the NF-κB p65/p50 proteins from the cell cytoplasm to the nucleus. To verify this hypothesis, immunofluorescence assays were performed. As expected, the protein levels of p50/p65 significantly decreased in a dose-dependent manner after ATL treatment (Fig. 6d). Thus, ATL-mediated inhibition of glioblastoma cell growth might be mediated by the inhibition of the translocation of NF-κB and p300 from the cell nuclei to the cytoplasm, which then further inhibited the expression of COX-2.

### ATL inhibits IKKβ activity by targeting the ATP binding site

**Fig. 7:**
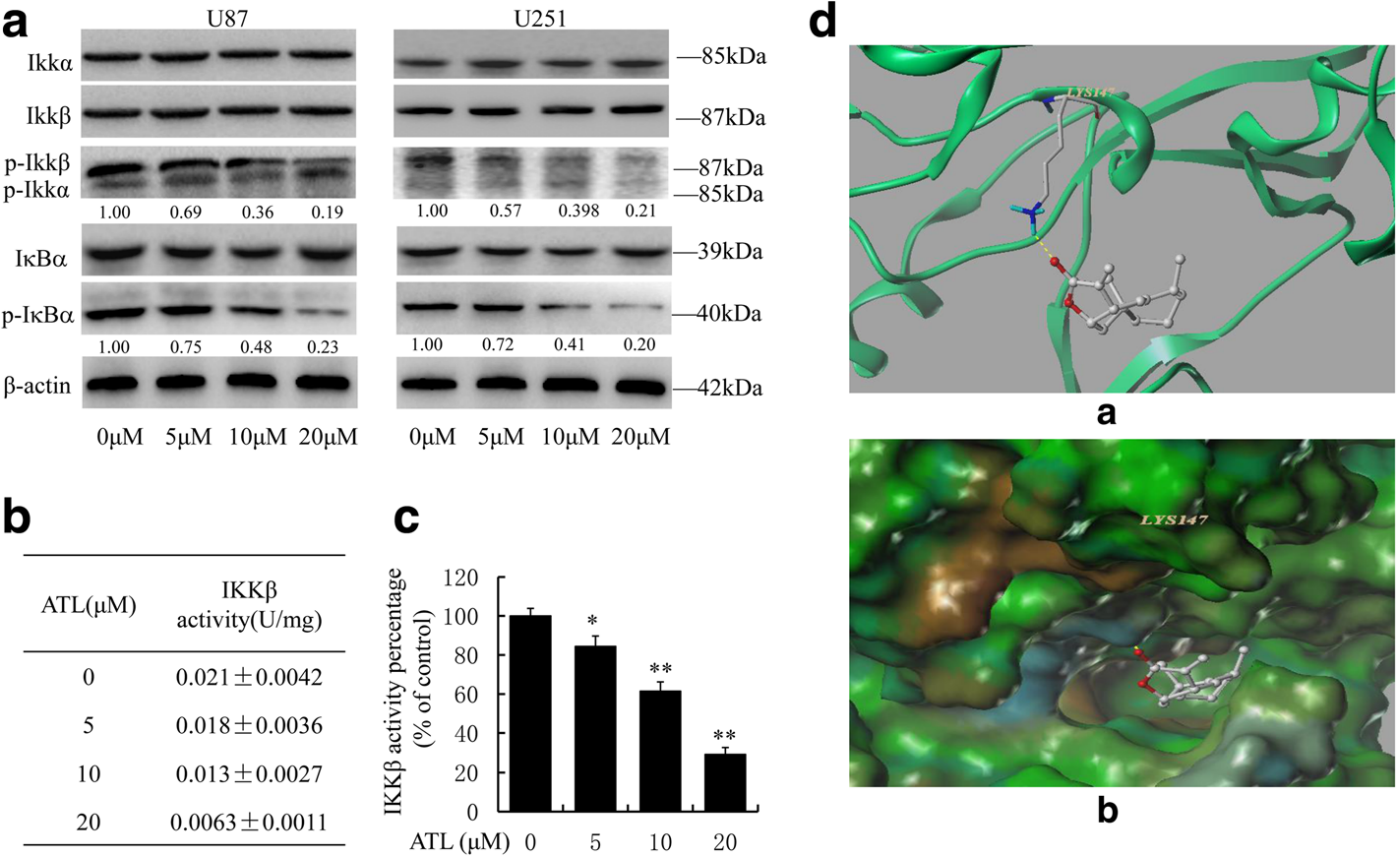
ATL suppresses IKKβ activity by targeting the ATP binding site. **a**: At 48 h after treatment, we observed the expression levels of IκB-α, p-IκB-α, IKKα/β, and p-IKKα/β by Western blotting in U87 and U251 cells. **b**-**c**: At 48 h after treatment, we also assessed IKKβ kinase activity in vitro using a cell IKKβ kinase activity spectrophotometry quantitative detection kit in U87 cells. The specific protocol was described in the “Materials and Methods” section, and the activity value and percentage were calculated using the provided formula. The results are represented as the mean ± SD of three experiments. *P < 0.05, **P < 0.01 vs. the DMSO-treated group. **d**: The best ranked position of ATL in the ATP binding site of IKKβ generated docking. (a) Interactions of ATL and IKKβ are delineated by the ribbon structure, hydrogen bonds are displayed as yellow dashed lines, and the participating amino acid residues are marked. (b) MOLCAD representation of the molecular lipophilic potential surface upon the bioactive position of ATL in the ATP binding site of IKKβ. The moieties are denoted as blue for hydrophilic, brown for lipophilic and green for neutral

Only activated NF-κB p50/p65 dimers are able to translocate to the nucleus and promote gene transcription [27]. However, the IKK complex is required for the activation of NF-κB, which is a major upstream kinase in the IκB-α canonical NF-κB signaling pathway. We, therefore, investigated whether ATL inhibited IKK activity to explore the potential molecular mechanisms. As shown in Fig. 7a, pretreatment with ATL significantly decreased the expression level of p-IκB-α and p-IKKβ in U87 and U251 cells but had little influence on the expression of p-IKKα and the overall expression of IKKα/β. Thus, ATL might suppress NF-κB activation by inhibiting IKKβ kinase activity because IKKβ is the major subunit that is responsible for the phosphorylation of IκB proteins.

Moreover, we assessed ATL-mediated inhibition of IKKβ kinase activity in vitro using a cell IKKβ kinase activity spectrophotometry quantitative detection kit. As shown in Fig. 7c, pretreatment with ATL could efficiently inhibit IKKβ kinase activity as follows: control group, 0.021 ± 0.0042 U/mg; ATL 5 μM group, 0.018 ± 0.0036 U/mg; ATL 10 μM group, 0.013 ± 0.0027 U/mg; and ATL 20 μM group, 0.0063 ± 0.0011 U/mg.

Furthermore, a computer molecular modeling assay was performed to simulate the interactions between ATL and IKKβ. The molecular docking studies predicted that ATL could bind to the ATP binding site of IKKβ. Specifically, as shown in Fig. 7d (a), ATL formed one hydrogen bond with the ATP binding pocket of the IKKβ kinase domain. The CO motif in the lactonic ring of ATL formed a hydrogen bond with the backbone NH of Lys147. The results of the MOLCAD surface modeling indicated that the lactone ring of ATL interacted with the residues at the entrance of the ATP-binding pocket to block the nucleotide recognition domain binding with ATP (Fig. 7d, b). Overall, IKKβ is a target site of ATL in the NF-κB signaling pathway to suppress COX-2 expression.

### ATL inhibits tumor xenograft growth in nude mice

**Fig. 8:**
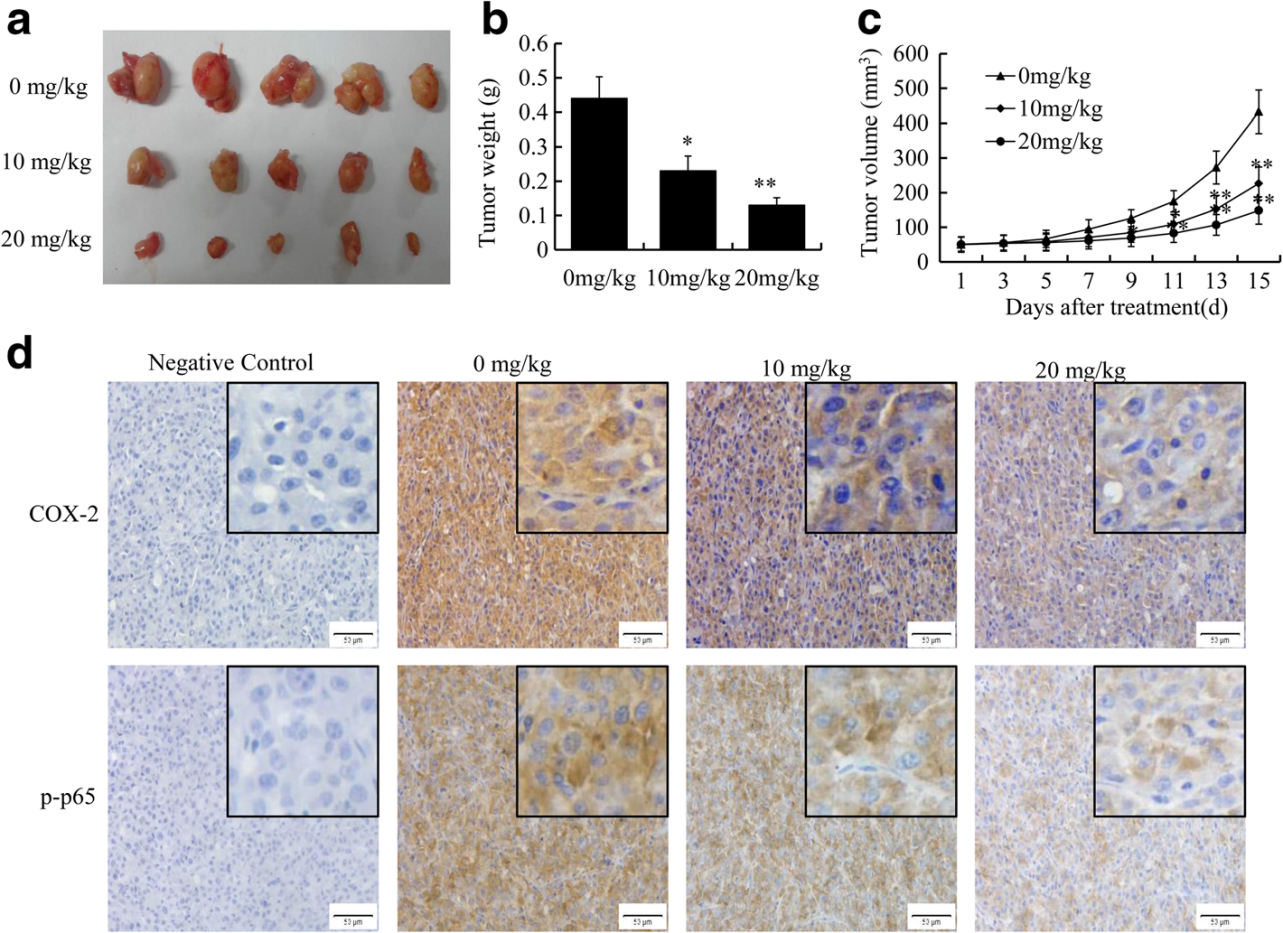
ATL suppresses the growth of tumor xenografts in nude mice. First, we established a subcutaneous transplant tumor model in nude mice by inoculating U87 cells. **a**: Photographic illustration of tumors from control and ATL-treated nude mice after 15 days of treatment. **b**-**c**: Tumor volumes and tumor weights. **d**: The protein expression of COX-2 and p-p65 in tumor samples by immunohistochemical analysis (original magnification, 400×)

Based on the results of the in vitro studies, we further explored the potential of ATL as a novel molecular therapeutic agent against tumor growth in nude mice. Compared with the control group, both the tumor volumes (Fig. 8a) and the tumor weights (Fig. 8b) in treated mice significantly decreased after the administration of 10 and 20 mg/kg/day of ATL for 15 days. The tumor inhibition rates (Fig. 8c) were 47.73 ± 9.32% in the 10 mg/kg group and 70.45 ± 13.33% in the 20 mg/kg group. No obvious toxic effects were detected in the mice that were treated with ATL. In addition, immunohistochemical staining was performed to examine the expression of COX-2 and p-p65 in vivo. As shown in Fig. 8d, ATL significantly reduced the expression of COX-2 and p-p65 in tumor tissues. Thus, ATL could inhibit the growth of xenografted U87 cells by down-regulating the expression of COX-2.

### ATL can penetrate the BBB

**Fig. 9:**
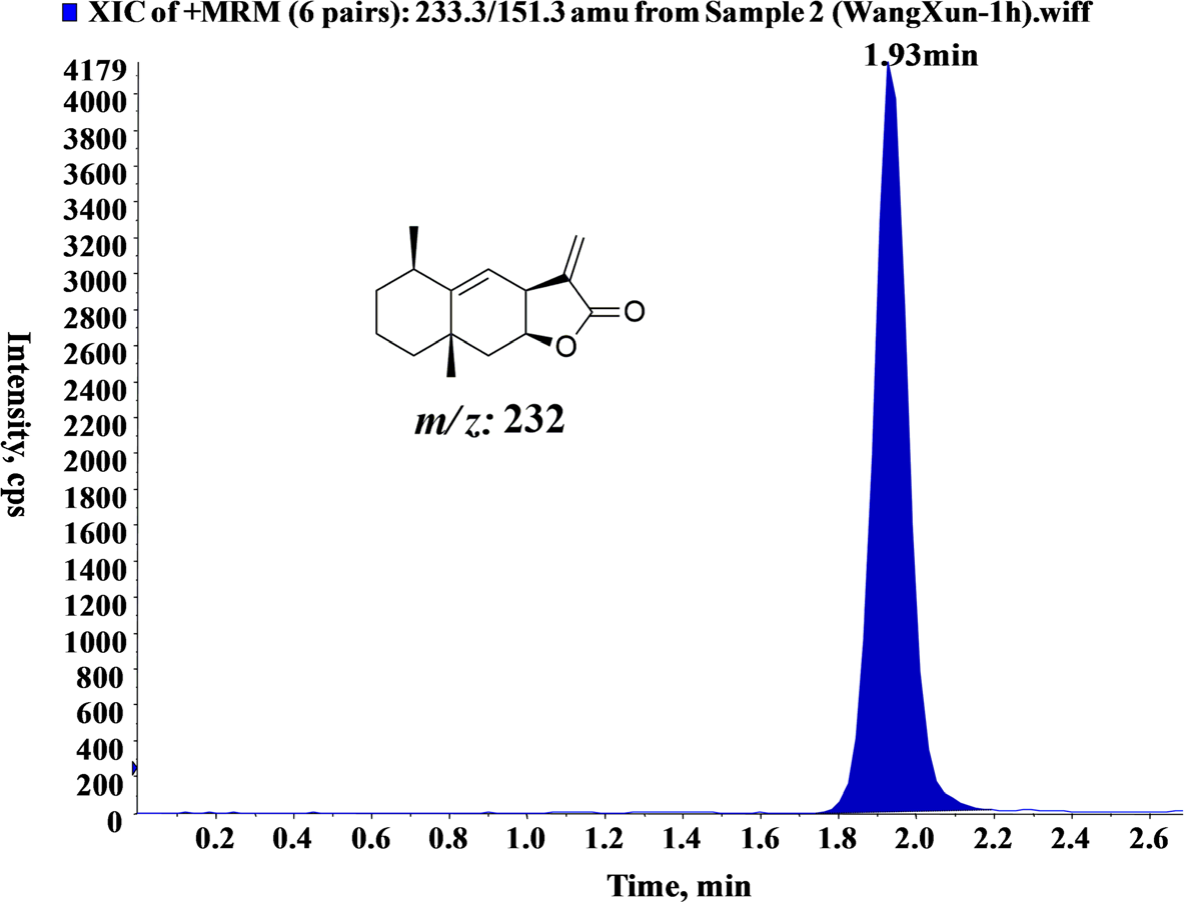
ATL can penetrate the BBB. LC-MS spectrum of cerebrospinal fluid sample in an SD rat model after pretreatment with ATL (1 h)

Subsequently, we determined whether ATL could traverse the BBB because the BBB is the greatest barrier for nearly 98% of small molecules from the blood into the brain [45]. In this study, the collected cerebrospinal fluid was assessed by an LC-MS/MS assay. As shown in Fig. 9, after the administration of ATL, a high chromatographic peak with Q1-Q3: 233.3-151.3 was observed at 1.93 min in the cerebrospinal fluid samples, which was consistent with the ATL standard sample. Therefore, we confirmed that ATL could penetrate the BBB, which might allow for a much more effective bioactivity in CNS diseases, particularly GBM.

## Discussion

GBM is the most refractory and palindromic CNS neoplasm. The main reasons for the poor clinical treatment effect in GBM are as follows: A. rapid proliferation; B. infiltrative growth; C. and the BBB. Therefore, it is essential to discover novel targeted therapeutic agents. In this study, we found that the natural sesquiterpene lactone compound ATL inhibited glioblastoma cell growth, and we explored the mechanism underlying its anti-tumor effects.

ATL, which is isolated from the Chinese herb *Inula helenium*, possesses multiple pharmacological activities, and its anti-tumor activity is highly attractive [32, 46, 47]; however, the pivotal molecules targeted by ATL remain unclear. In this study, we uncovered one anti-tumor mechanism by which ATL inhibited glioblastoma cell growth and induced cell apoptosis by inhibiting the NF-κB/COX-2 signaling pathway and activating the cyt c/caspase-dependent apoptotic pathway. Furthermore, ATL acted as an inhibitor of IKKβ activity by targeting the ATP-binding site to suppress COX-2 expression in glioblastoma cells.

The expression of COX-2 is positively correlated with the degree of malignancy in the glioma and is negatively correlated with the prognosis. In this study, ATL significantly inhibited COX-2 expression at both the protein and mRNA levels. We selected celecoxib (CB) as a positive drug treatment because CB is a classical and potent commercial COX-2 inhibitor. The combination of CB and ATL did not further alter cell viability; thus, we think that ATL suppressed growth by partially inactivating COX-2 signaling. Moreover, the cell viability IC_50_ values after ATL treatment in U87 and U251 cells were 20.24 and 16.33 μM, which were significantly lower than those of CB (135.27 and 120.32 μM). Thus, ATL, which is a natural small molecule inhibitor, is comparable to CB but is more effective, safe and affordable.

The transcription factor NF-κB, which is a cis-acting element of the COX-2 transcriptional regulatory sequence, is involved in the regulation of a variety of inflammatory mediators. Activated NF-κB binds the COX-2 promoter region and initiates COX-2 transcription. During the activation of NF-κB, the transcription coactivator p300 can bind the NF-κB p65/p50 dimer by acetylation and thus enhance the transcriptional activity of COX-2. Our study demonstrated that ATL could inhibit the nuclear translocation of the NF-κB p65/p50 dimer, decrease the recruitment of p300, and thus suppress their binding to the COX-2 promoter region, thereby blocking COX-2 transcriptional activity and down-regulating the expression of COX-2.

The IKKs are key regulators in the NF-κB signaling pathway, and we demonstrated that ATL could specifically inhibit IKKβ enzyme activity via an in vitro kinase assay. Furthermore, computational docking analysis suggested that ATL occupied the entrance hydrophobic pocket in the ATP-binding site of IKKβ. In this modeling analysis, ATL was located well in the ATP binding site and interacted with residue Lys147 at the entrance of the ATP-binding pocket. Our results suggested that ATL might block the nucleotide recognition domain binding with ATP as a reversible inhibitor. These findings are consistent with our experimental results. Hydrophobic interactions should be emphasized because the ATP binding pocket is a narrow and hydrophobic region. ATL may attenuate the transcriptional activity of NF-κB at least in part by abrogating the activity of IKKβ.

Infiltrative growth is another major cause of refractory GBM. Migration and invasion are two important prerequisites for infiltrative growth, and degradation of the extracellular matrix is a key step. MMP-2 and MMP-9 are gelatinases that are important members of the MMP family, which plays a significant role in breaking through the extracellular matrix of cells [48]. MMP-2 and MMP-9 are negatively correlated with the prognosis of glioma patients [49]. Intriguingly, our study illustrated that ATL could inhibit the migration and invasion of GBM cells and significantly decrease MMP-2 and MMP-9. As MMP protein is expressed in tumor cells and blood vessels, and angiogenesis is an important link in the invasion and metastasis of malignant tumors, the inhibitory properties of ATL implies that metastasis and invasion may be another target for ATL to suppress tumor growth or angiogenesis, and the underlying mechanism requires further investigation.

Furthermore, the BBB is a major limitation that reduces the efficacy of anti-cancer drugs in the treatment of GBM patients [50]. Studies have confirmed that the cerebrospinal fluid brain barrier is one of the most imperfect barriers in the BBB and can allow cerebrospinal fluid and the extracellular fluid of brain tissue to communicate with each other [45]. Therefore, once a substance enters the cerebrospinal fluid from the blood, it can freely diffuse into the brain tissue; thus, we can detect the drug content in the cerebrospinal fluid, which is an important method for evaluating drug entry into the brain tissue [51]. In our study, ATL was detected by LC-MS/MS analysis in cerebrospinal fluid collected from living rats. Thus, ATL could pass through the BBB, which fulfilled a prerequisite for ATL in the treatment of CNS diseases.

## Conclusions

**Fig. 10:**
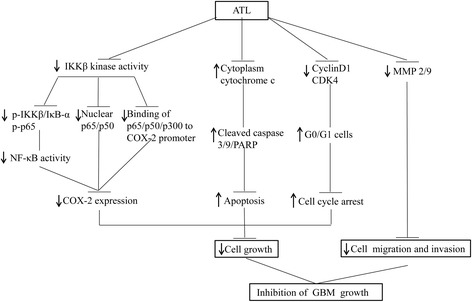
Schematic showing that ATL effectively inhibits GBM growth. ATL suppressed IKKβ kinase activity and then inhibited the phosphorylation of IKKβ, IκBα and the p65 proteins, promoted the translocation of NF-κB from the cell nuclei to the cytoplasm and attenuated the binding of NF-κB and p300 to the COX-2 promoter, thereby reducing the expression of COX-2. In addition, ATL promoted the release of cyt c from the mitochondria to the cytoplasm and suppressed the expression of cyclin D1, CDK4 and the MMP2/9 proteins

In conclusion, ATL can effectively inhibit GBM cell growth and enhance apoptosis. Furthermore, ATL suppresses the expression of COX-2 by suppressing IKKβ activity by targeting the ATP-binding site, thereby inhibiting the translocation of the NF-κB p65/p50 proteins from the cell cytoplasm to the nucleus and abrogating NF-κB binding and p300 recruitment on the COX-2 promoter (Fig. 10). In addition, ATL can penetrate the BBB. These findings provide strong evidence for the potential of ATL as a small molecule natural inhibitor with a broad potential for clinical applications in CNS diseases, such as GBM treatment.
