# Correction to: Alantolactone, a natural sesquiterpene lactone, has potent antitumor activity against glioblastoma by targeting IKKβkinase activity and interrupting NF-κB/COX-2-mediated signaling cascades

**DOI:** 10.1186/s13046-020-01699-4

**Published:** 2020-09-23

**Authors:** Xun Wang, Zhenlong Yu, Chao Wang, Wei Cheng, Xiangge Tian, Xiaokui Huo, Yan Wang, Chengpeng Sun, Lei Feng, Jinshan Xing, Yulong Lan, Dongdong Sun, Qingjuan Hou, Baojing Zhang, Xiaochi Ma, Bo Zhang

**Affiliations:** 1 Department of Neurosurgery of the Second Affiliated Hospital, College of Pharmacy, Institute of Cancer Stem Cell, Dalian Medical University, Dalian, China; 2 Department of Neurosurgery, the Third People’s Hospital of Dalian, Non-directly Affiliated Hospital of Dalian Medical University, Dalian, China

**Correction to: J Exp Clin Cancer Res 36, 93 (2017)**

**https://doi.org/10.1186/s13046-017-0563-8**

Following publication of the original article [1], the authors identified errors in Fig. 3c and Fig. 7a. The lane protein of β-actin for U251 cells in Fig. 3c and the IKKα for U87 and IκBα for U251 in Fig. 7a were mis-uploaded. The correct figures are given below. The authors declare that these corrections do not change our results or conclusions of this article and apologize for any confusion that have caused.

**Fig. 3:**
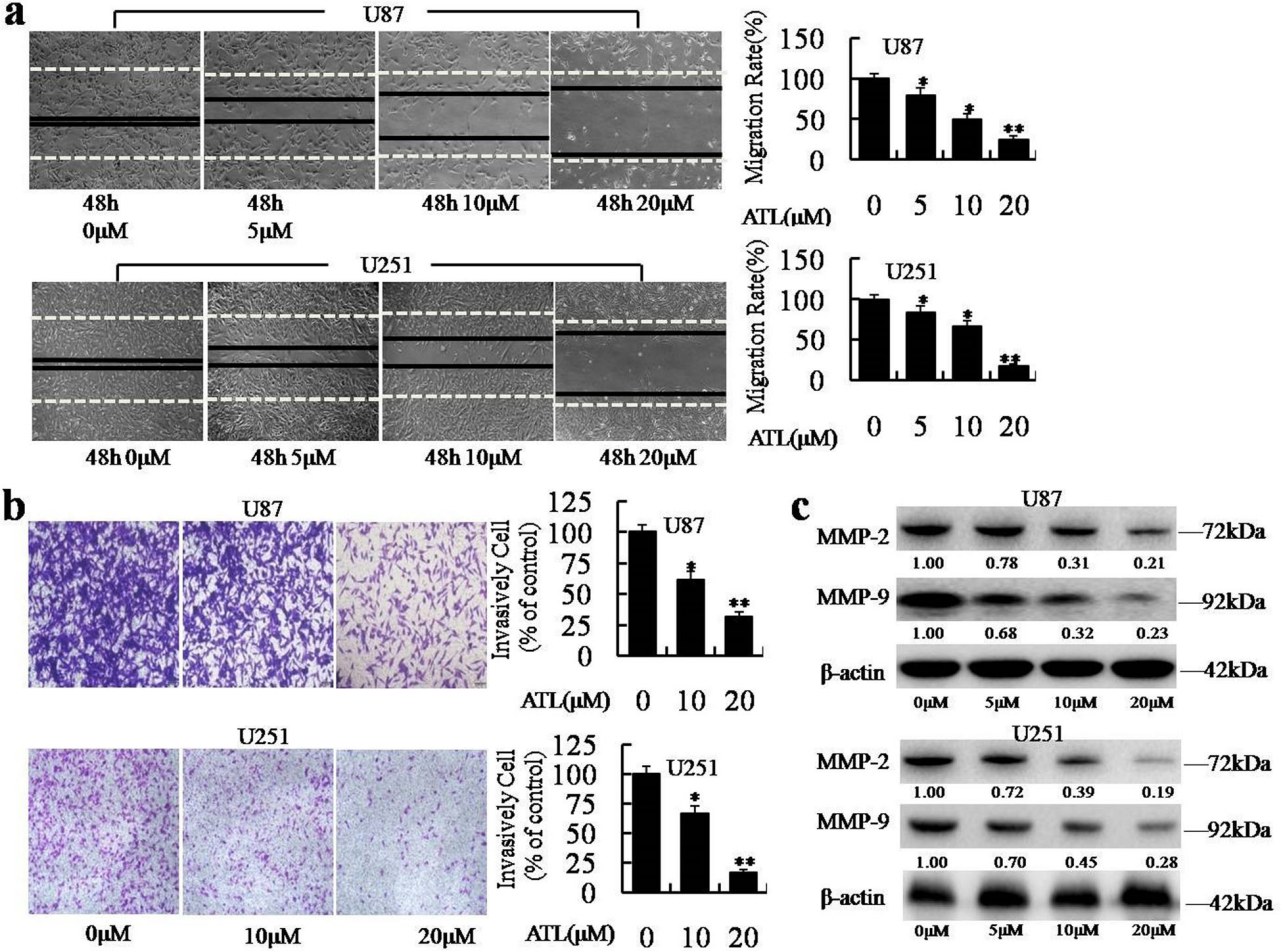
ATL inhibits cell migration and invasion. **a**: Cell migration was analyzed using a wound-healing assay in U87 and U251 cells as described in the “Materials and Methods” section (original magnification, 100×); then, the migration rate was calculated. **b**: ATL suppresses cell invasion in the Transwell assay. The two cell types were plated in Matrigel pre-coated Transwell chamber. Cells that have passed through to the bottom of the membrane were counted (original magnification, 100×). **c**: MMP-2 and MMP-9, which are invasive marker proteins, were detected by Western blotting in the two cell lines. Three independent experiments were performed. *P < 0.05, **P < 0.01, vs. the DMSO-treated group

**Fig. 7:**
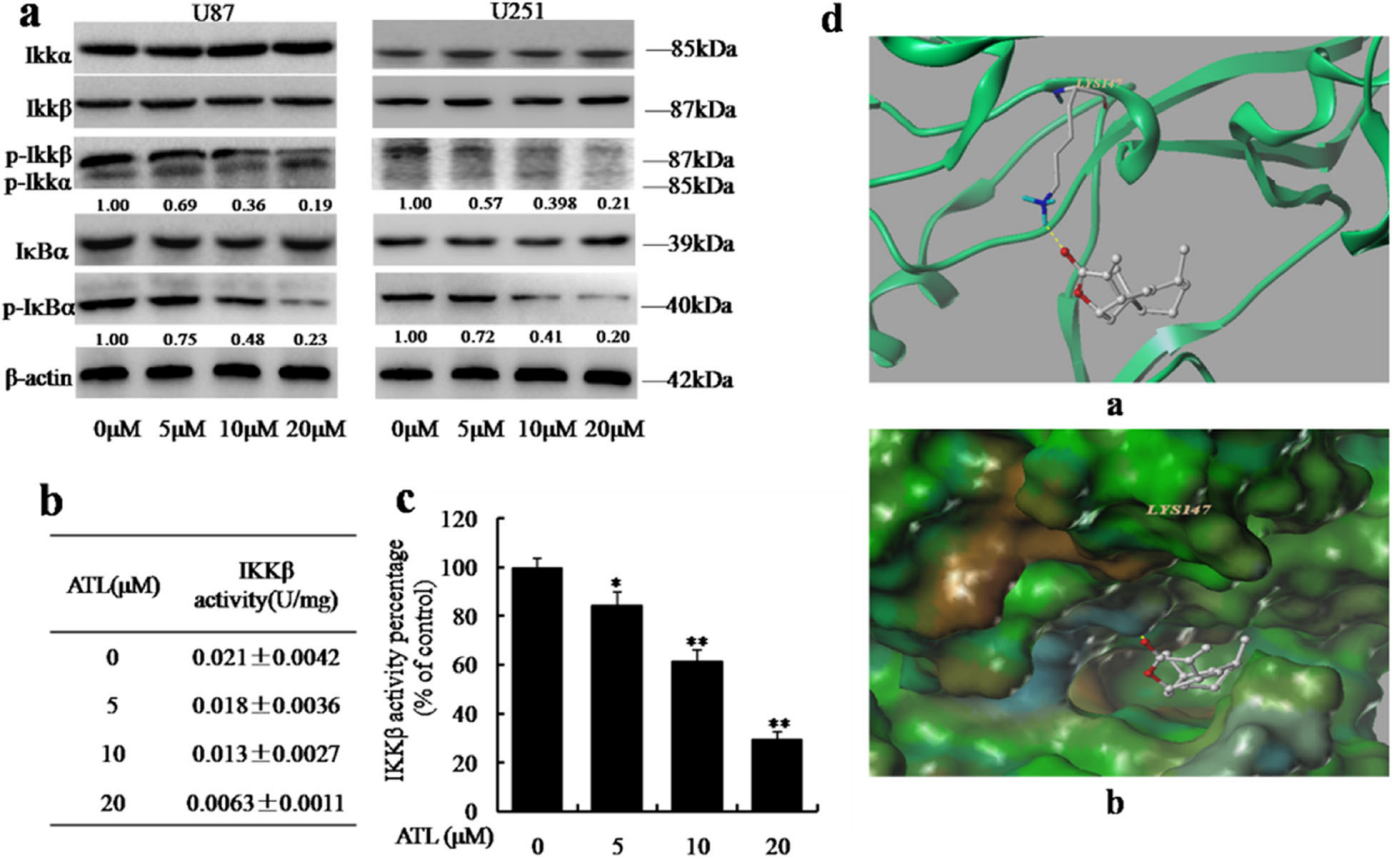
ATL suppresses IKKβ activity by targeting the ATP binding site. **a**: At 48 h after treatment, we observed the expression levels of IκB-α, p-IκB-α, IKKα/β, and p-IKKα/β by Western blotting in U87 and U251 cells. **b**-**c**: At 48 h after treatment, we also assessed IKKβ kinase activity in vitro using a cell IKKβ kinase activity spectrophotometry quantitative detection kit in U87 cells. The specific protocol was described in the “Materials and Methods” section, and the activity value and percentage were calculated using the provided formula. The results are represented as the mean ± SD of three experiments. *P < 0.05, **P < 0.01 vs. the DMSO-treated group. **d**: The best ranked position of ATL in the ATP binding site of IKKβ generated docking. (a) Interactions of ATL and IKKβ are delineated by the ribbon structure, hydrogen bonds are displayed as yellow dashed lines, and the participating amino acid residues are marked. (b) MOLCAD representation of the molecular lipophilic potential surface upon the bioactive position of ATL in the ATP binding site of IKKβ. The moieties are denoted as blue for hydrophilic, brown for lipophilic and green for neutral

## References

[1] Wang X, Yu Z, Wang C (2017). Alantolactone, a natural sesquiterpene lactone, has potent antitumor activity against glioblastoma by targeting IKKβ kinase activity and interrupting NF-κB/COX-2-mediated signaling cascades. J Exp Clin Cancer Res.

